# 2340 A robust spatial normalization pipeline for individuals with focal cortical lesions

**DOI:** 10.1017/cts.2018.41

**Published:** 2018-11-21

**Authors:** Andrew DeMarco, Peter Turkeltaub

**Affiliations:** Georgetown - Howard Universities

## Abstract

OBJECTIVES/SPECIFIC AIMS: To develop a robust spatial normalization pipeline for brains of individuals with focal cortical lesions. METHODS/STUDY POPULATION: Individuals with chronic focal cortical lesions from stroke. RESULTS/ANTICIPATED RESULTS: We have developed a robust spatial normalization pipeline for brains of individuals with focal cortical lesions, and demonstrate how the pipeline overcomes obfuscated neuroanatomy to yield both consistent and excellent results. DISCUSSION/SIGNIFICANCE OF IMPACT: Our robust normalization pipeline will enable group analyses of individuals with cortical lesions with greater spatial precision. This greater spatial precision will improve answers to questions about functional localization in the brain, and ultimately allow translation of findings from neuroimaging studies in individuals with cortical lesions to the clinic.

